# A non-invasive Ayurveda management of venous leg ulccer- A case report

**DOI:** 10.1016/j.jaim.2024.101073

**Published:** 2025-03-28

**Authors:** Archana Muraleedharan, Sree R. Nair, P.N. Rajeshwari

**Affiliations:** Department of Shalya Tantra, Amrita School of Ayurveda, Amrita Vishwa Vidyapeetham, Kollam, Kerala, India

**Keywords:** Ayurveda, Case report, Lipodermatosclerosis, *Patradāna*, Varicose ulcer

## Abstract

Venous leg ulcer (VLU) is a severe complication of chronic venous insufficiency that commonly affects older individuals. It is a long-standing consequence of venous insufficiency and accounts for 70%–80% of lower limb ulcers seen in outpatient departments. These ulcers are often unresponsive to treatment and can significantly impact patients' quality of life, causing pain, sleep disturbances, and decreased mobility. Managing varicose leg ulcers is a significant challenge for physicians.A 55-year-old male presented with symptoms including pain, stiffness, discoloration, swelling in the left lower limb, and a history of non-healing ulcer. The patient was treated with *Patradana* using leaves of *Ricinus communis* (*Eranda patra*) and *Grihadhumadi lepa* along with oral medications such as *TriphalaGuggulu* and *Gandhaka Rasayana*. Each day, the change was carefully observed and documented, revealing a notable improvement by the end of the treatment (10 days). The pain, swelling, and stiffness exhibited significant reduction, accompanied by wound healing after the first follow-up of 14 days. The complete wound healing was achieved and quality of life showed improved. The patient has beenunder observation and has not reported any recurrence of ulcer in the 4 months of follow up.

## 1 Introduction

**Fig. 1:**
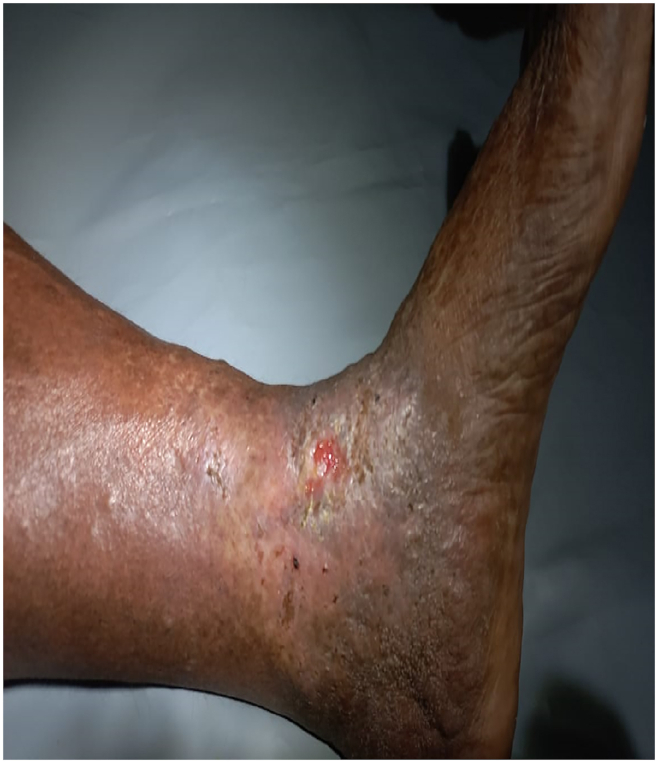
The pictorial visualization of Ulcer on Day 1.

**Fig. 2:**
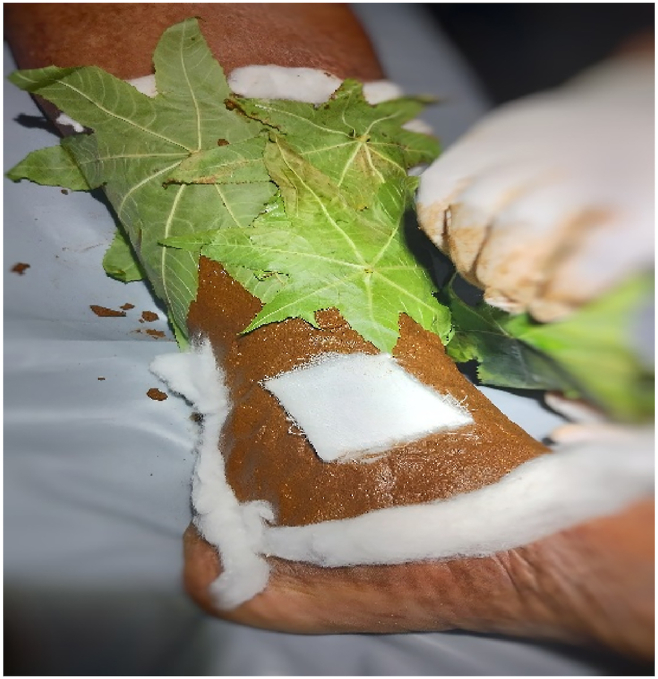
The picture of procedure *Patradāna*.

**Fig. 3:**
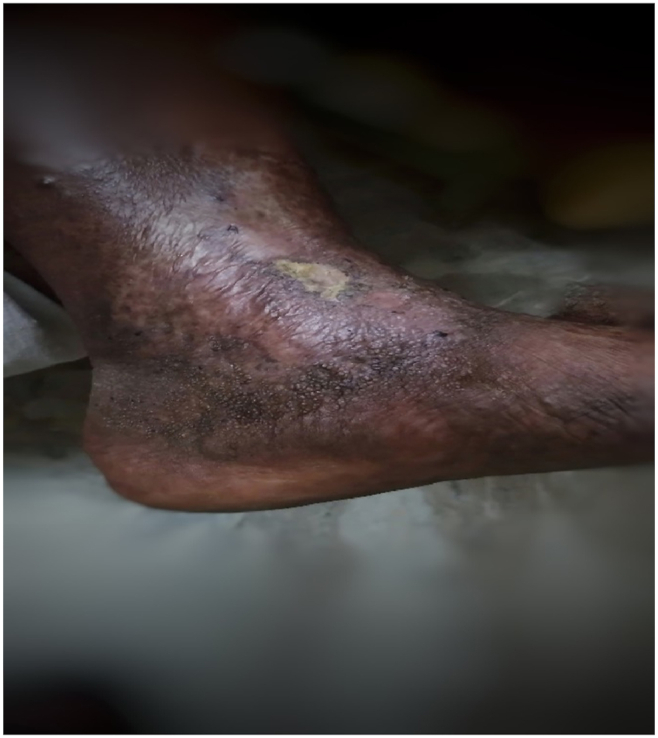
The ulcer of second follow up. (36th day from initial visit).

An ulcer is a break in the continuity of the covering epithelium – skin or mucous membrane [1]. If this ulcer is associated with insufficiency of venous circulation, it is called a venous ulcer/varicose ulcer/stasis ulcer/gravitational ulcer. Basic cause of this ulcer is abnormal venous hypertension in the lower third of the leg, ankle and dorsum of the foot. In normal conditions, calf muscle contraction and intraluminal valves promote forward flow while preventing blood reflux. When retrograde flow, obstruction, or both together leads to chronic venous hypertension, and it also act as a reason for blood stagnation and then it is responsible for the dermatologic and vascular complications that culminate in the formation of a venous leg ulcers. Venous leg ulcers are open lesions of the lower limb and represent between 60 and 80% of all leg ulcerations that occur in the presence of venous disease. Venous leg ulcers are chronic wounds characterized by slow healing and high recurrence [2]. These ulcers represent the most advanced form of chronic venous disorders like varicose veins and lipodermatosclerosis. Predisposition factor for development of VLUs include old age, female sex, overweight, trauma, immobility, congenital absence of veins, deep vein thrombosis (DVT), phlebitis, and factor V Leiden mutation [3]. Varicose ulcer results in reduced mobility, significant financial implications, and poor quality of life. The management of varicose ulcers poses a difficult challenge for many medical professionals. This is especially true when symptoms are intermittent or recurring. Therefore, an efficient protocol for managing varicose ulcer was essential and thereby increasing the quality of life of patients (see Table No.1, Table No. 2, Table No. 3, Fig. 1, Fig. 2, Fig. 3).

**Table No.1:** Examination of left lower limb

INSPECTION	PALPATION
Swollen left lower limb	Dorsalis pedis – was palpable
Brownish black discolouration – below calf (Left)	Warmth - present, pitting edema
Lipodermatosclerosis	Hardening of skin – below and around calf region
Ulcer of medial malleoli	Tenderness – present
Pale granulation tissue with in the ulcer

**Table No. 2:** Examination of ulcer

Size, Site and Dimension	Solitary, above to left medial malleoli
	Length – 3.5 cm
	Breadth – 2.5 cm
	Depth – 0.5 cm
Odour	No Foul odour noticed
Discharge	Serous discharge
Edge	Sloping, irregular
Shape	Vertical oval
Margin	Irregular
Depth	Shallow
Surrounding skin	Eczematous and pigmented

Varicose veins can correlate with *sirajagranthi*, which falls under the category of - a disease with no complete cure. The main defect is typically located at the Sapheno-Femoral-Junction or Sapheno-Popliteal-Junction. This is due to venous reflux or obstruction, both of which lead to poor venous return and venous hypertension; which is the main pathology. Over time, this may lead to the formation of ulcers. The ulcers may be associated with Lipodermatosclerosis, Atrophie Blanche and sometimes pitting edema. These ulcers can be classified as C_5_ if they are healed or C_6_ if they are active ulcers. A new classification, C_6r_, has been introduced for recurring active ulcers. In Ayurveda, this type of non-healing ulcer would fall into the category of *dustavrana* (septic non-healing ulcer). The Ayurvedic management includes; *Lekhana* (scraping), *Kshara karma* (alkali therapy), and *rakta mokshana* (bloodletting therapy). Classical text, *Sushruta samhita*, explains 60 different treatment modalities in wound management [4]. *Patradana* is one such modality in which leaves are applied over ulcer. From these insights, trying to explore Ayurvedic wound healing methods in Varicose ulcer and also focused on normalisation or improvement of vein haemodynamics, improvement or elimination of congestion pain (heaviness, tension, heat, pain) and persistent edema, reduction of the recurrence rate of ulcers and other forms of trophic disturbances.

## 2 Patient information

A 55-year-old male patient came to Shalya tantra OPD of Amrita Ayurveda Hospital, Karunagappally. He complained of pain, swelling, discoloration, and stiffness of the left lower limb with a non-healing ulcer on the medial malleoli of the same leg since 2016. As per CEAP classification this patient condition corresponds to C3,4,6, E_s_, A_s,_ P_r,o_ [5]. He had received some relief initially, but the condition eventually worsened. Patient had a history of varicose vein since 15 years and history of known and treated case of CA bladder neck (2010). He worked as an Operation Theatre assistant for the past 25 years for which he had a history of prolonged standing.

There was no history of diabetes mellitus, hypertension or any other systemic disease. There was no relevant family history. Patient had no history of smoking, tobacco chewing or alcohol consumption.

## 3 Clinical findings

Patient was moderately build and well-nourished; found with pitting edema, discolouration and a non-healing ulcer over medial malleolus on left lower limb. Patient was conscious and well oriented at the time of admission. All vitals were within normal limits during the stay in the hospital. Other systemic examination revealed normalcy.

### 3.1 Local examination

**Table No. 3:** Timeline of events.

Days	Wound size (Length X Breadth X Depth) in mm	Bates Jensen wound assessment scale	Clinical observation and recordings
Day 1	35 X 25 X 5	• Size – 2 • Exudate type - 4 • Edge - 3 • Depth - 3 • Skin colour surrounding wound - 4	• Assessemnet of Nature of The Ulcer • Starting The Patradana Treatment with Internal Medications (Day 1 to Day 10)
Day 2	35 X 24 X 5		
Day 3	34 X 22 X 5		
Day 4	33 X 20 X 3		
Day 5	31 X 17 X 2		
Day 6	30 X 15 X 1		
Day 7	27 X 13 X 1		
Day 8	22 X 10 X 1		
Day 9	19 X 8 X Less than 1mm		
Day 10	14 X 5 X 0 (Almost the granulation tissue had covered)	• Size – 2 • Exudate type - 1 • Edge - 2 • Depth – 2 • Skin colour surrounding wound - 2	Discharge Of the Patient Along with Internal Medicines Healthy Granulation Tissue Present
First Follow Up (22nd Day from Initial Visit/12 days from discharge)	The wound was in healing stage and scab was remaining; the pigmentation reduced. Swelling absent. No discharge, no foul smell.		The Wound Was in Healing Stage Along with Internal Medicines
Second Follow Up (36th Day from Initial Visit/12 days from first follow up)	The wound healed completely; the surrounding skin was healthy.	• Size – 1 • Exudate type - 0 • Edge - 1 • Depth – 1 • Skin colour surrounding wound −1	Complete Healing Wound and Stoppage of All Medications

## 4 Diagnostic assessment

Patient was a known case of Varicose vein since 15 years and ulcer present over medial malleolus which is less painful in nature. The presenting complaints and history are suggestive of Venous leg ulcer without any challenges in diagnosis.

## 5 Therapeutic intervention

The patient was under both internal medications and external therapy. The internal medicines.

1 *TriphalaGuggulu* 500 mg twice daily before food
2 *Gandhaka Rasayana* 500 mg twice daily After food)

The external therapy include *Patradana*; using *Grihadhumadi lepah choornam* and *Eranda patra* for 7 consecutive days. And advised the patient with daily cleaning and dressing after discharge from the hospital.

• *Patradana* Procedure:

The area was cleaned with antiseptic solution. The wound was covered with sterile pad. The *Grihadhumadi lepa* was applied all around the wound; below calf. Then *Ricinus communis* leaves once autoclaved for 3 mins at 100 °C were spreaded over the applied *lepa*. A sterile gauze roll was wrapped to retain the *lepa* in position for 4 hours. After that the *lepa* was removed and clean the wound with normal saline. The wound was kept closed with sterile gauze pad till next day.

### 5.1 Diet

Diet plays an important role in ulcer management. The nourishment has to be provided in association with wound healing; hence, spicy, sour, salty foods were avoided for him. He was restricted for day sleep, walking in sun, waking up late night. He was advised with brisk walk in the evening without any exertion.

## 6 Assessment criteria

Wound size was measured using ruler and daily assessments recorded without fail. (See table No.3).

## 7 Follow UP and outcomes

Daily observations were noted and recorded. Once the patient was discharged from the hospital he was advised to revisit the hospital after 2 weeks follow up. After 4weeks of follow up there had a complete wound healing. The outcomes are noted in Table No.3.

## 8 Timeline of the study

Refer Table No.3.

## 9 Discussion

Treating a venous ulcer is a challenge for many physicians because of its ‘on and off’ symptoms. The above detailed case was one such; which was treated very carefully as he was a cancer survivor. Non healing Venous ulcer may tend to become cancerous as because of its nature. Here the patient was effectively treated with *pana*; one among the 60 principles in managing an ulcer by *Sushruta Samhita*.

The *pana* was done using *Gadi lepa* and leaves of *Ricinus communis*. The clinical features of VLU corresponds to the clinical features of *vata rakta* hence this combination was selected. *Gadi lepa* contains *Grhaduma, Vha, Kushtha,Shatahva,Haridra,* and *Daruharidra*, they are *pitta rakta* hara and *Srotoshoodhana,* *Shulahara* in nature. *Grihadhumadi lepa* having *kaphavatahara karma* along with *Shulahara* *Shophahara* and *Srotoshodhana* action. *Grihadhumadi lepa* might have reduced the symptoms like swelling, hardening of the skin and pain associated with it.

The leaves of *Ricinus communis* has been stated in patradāna concept of Sushrutha Chikitsa [6]; since in this patient locally manifestations of Vata-dosha-aggravation were evident we preferred to choose these leaves. The leaves were collected freshly possessing the quality of a good leaves was selected. Then it was autoclaved for 3 mins at 100 °C. *Patradana* is indicated when there is presence of static ulcer (*sthira*) with less of muscles (*alpamamsa*), not healing due to dryness *(rukshata*) [7]. The above mentioned case was having such features hence this treatment was selected. The aforesaid *lepa*, and leaves of *Ricinus communis* was together helpful in reducing the symptoms like pain, swelling and pigmentation. Once the circulation improved it accelerated the wound healing process.

Internally the patient was given with *Triphala* *guggulu* and *Gandhaka* *rasayana*, inorder to prevent infection and aid in healing process. *Triphala* *guggulu* and *Gandhaka rasayana* might have shown its anti-inflammatory, anti-microbial action and wound healing property in this case of VLU.

In total the combined action of both internal and external medications along with proper diet would have driven a non-healing ulcer to its healing pathway. The ulcer got completely healed around 35 days with a healing rate of 0.8 cm^2^/day. As the ulcer was healing all the other symptoms like hardening of skin, swelling and discolouration and serous discharge got disappeared; these points out the complete healing of the Ulcer.

The patient was under observation for right knee joint pain and he does not have complaints of relapse of ulcer for about 4 months (January 14, 2024)

## 10 Conclusion

The study shows a noteworthy change in the patient's condition. The *Pana* promoted the phases of wound healing by improving circulation. Each day remarkable changes were noticed and with reduced symptoms. Progression of the wound was arrested and in tandem with Ayurvedic oral medication, the wound healed. The aim of the treatment was to make healing faster. The treatment opted was based on the *dsha* involved. *Pana* adopted was to accelerate healing; reduction in swelling and hardening of skin; decreased pain along with the improvement in circulation was achieved. The strength of the case can be understood as the VLU was manageable with non-invasive ayurvedic medicines without any relapse. Managing VLU is haunting for many physicians due to its nature of relapse; this case protocol can be replicated for many such VLU cases and *patradana* procedure can be standardized.

## Patient perspective

"Dear Doctor, As you know that I had a non-healing ulcer of my leg, for which I came here for the first time. As the treatment I received from here was so satisfied because the ulcer got healed completely with a short duration for which I was suffering from 2016. I was not able to go out of my house because of the comments and suggestions for this ulcer. Now I'm glad that I could go out freely and I cured completely from the ulcer. As per your advice I'm continuing the exercises. I'm thankful to the entire team of doctors and staffs."

## Informed consent

Written informed consent collected from the patient for the purpose of publication of this case report.

## Author contribution

Rajeshwari P N: Conceptualization, Methodology/study design, software, validation, formal analysis, resources, data curation, review and editing, visualization, supervision.

Archana Muraleedharan: Conceptualization, Methodology/study design, software, validation, investigation, resources, writing-original draft, review and editing, visualization.

Sree R. Nair: Conceptualization, Methodology/study design, software, validation, investigation, resources, writing-original draft, review and editing, visualization.

## Data statement

Data supporting the findings of this study are accessible within the article, and raw data are obtainable from the corresponding author upon reasonable request.

## Declaration of generative AI in scientific writing

During the preparation of this work, the authors used [Chat gpt] to improve the sentences. After using this tool/service, the authors reviewed and edited the content as needed and took full responsibility for the content of the publication.

## Declaration of competing interest

The authors declare the following financial interests/personal relationships which may be considered as potential competing interests:

Dr. Rajeshwari P N reports financial support was provided by 10.13039/100009526Amrita Vishwa Vidyapeetham Amrita School of Ayurveda. The other authors declare that they have no known competing financial interests or personal relationships that could have appeared to influence the work reported in this paper.

## References

[1] Das S. (2018).

[2] Probst S., Weller C.D., Bobbink P., Saini C., Pugliese M., Skinner M.B. (2021). Prevalence and incidence of venous leg ulcers—a protocol for a systematic review. Syst Rev [Internet].

[3] Vasudevan B. (2014). Venous leg ulcers: pathophysiology and classification. Indian dermatology online journal.

[4] Srikantha Murthy K.R. (2017).

[5] Vijayasankar Rajathi, Bhavani R., Jiji Wiselin (2019). Varicose ulcer(C6) wound image tissue classification using multidimensional convolutional neural networks. Imag Sci J.

[6] Srikantha Murthy K.R. (2017).

[7] Srikantha Murthy K.R. (2017).

