# Supplementary material for: A non-invasive Ayurveda management of venous leg ulccer- A case report
Source: J Ayurveda Integr Med. 2025 Mar 28;16(2):101073. doi: 10.1016/j.jaim.2024.101073 (PMC11994297; doi:10.1016/j.jaim.2024.101073)
Supplement: Multimedia component 1 [file mmc1.docx]

**TIMELINE OF THE STUDY**

Day 1

Assessemnet Of Nature of The Ulcer

Day 1- 10

Starting The *Patradāna* Treatment with Internal Medications

Day 10

Discharge Of the Patient Along with Internal Medicines

Healthy Granulation Tissue Present

First Review

The Wound Was in Healing Stage Along with Internal Medicines

22^nd^ Day from Initial Visit

Second Review

Complete Healing Wound and Stoppage of All Medications

36^th^ Day from Initial Visit
