# Supplementary material for: A non-invasive Ayurveda management of venous leg ulccer- A case report
Source: J Ayurveda Integr Med. 2025 Mar 28;16(2):101073. doi: 10.1016/j.jaim.2024.101073 (PMC11994297; doi:10.1016/j.jaim.2024.101073)
Supplement: Multimedia component 2 [file mmc2.docx]

Bates Jensen wound assessment tool

| Item | Assessment |
| --- | --- |
| Size | 1 = Length x width <4 cm  2 = Length x width 4--<16 cm  3 = Length x width 16.1--<36 cm  4 = Length x width 36.1--<80 cm  5 = Length x width >80 cm |
| Exudate type | 1= None  2 = Bloody  3 = Serosanguineous: thin, watery, pale red/pink  4 = Serous: thin, watery, clear  5 = Purulent: thin or thick, opaque, tan/yellow, with or without odour |
| Edge | 1 = Indistinct, diffuse, none clearly visible  2 = Distinct, outline clearly visible, attached, even with wound base  3 = Well-defined, not attached to wound base  4 = Well-defined, not attached to base, rolled under, thickened  5 = Well-defined, fibrotic, scarred or hyperkeratotic |
| Depth | 1 = Non-blanchable erythema on intact skin  2 = Partial thickness skin loss involving epidermis &/or dermis  3 = Full thickness skin loss involving damage or necrosis of subcutaneous  tissue; may extend down to but not through underlying fascia; &/or  mixed partial & full thickness &/or tissue layers obscured by  granulation tissue  4 = Obscured by necrosis  5 = Full thickness skin loss with extensive destruction, tissue necrosis or  damage to muscle, bone or supporting structures |
| Skin colour Surrounding wound | 1 = Pink or normal for ethnic group  2 = Bright red &/or blanches to touch  3 = White or grey pallor or hypopigmented  4 = Dark red or purple &/or non-blanchable  5 = Black or hyperpigmented |
